# Progress in kidney transplantation: The role for systems immunology

**DOI:** 10.3389/fmed.2022.1070385

**Published:** 2022-12-16

**Authors:** Aileen C. Johnson, Juliete A. F. Silva, Steven C. Kim, Christian P. Larsen

**Affiliations:** Department of Surgery, School of Medicine, Emory University, Atlanta, GA, United States

**Keywords:** kidney transplantation, rejection, graft failure, eplet, systems biology, multi-omics, immunopeptidome

## Abstract

The development of systems biology represents an immense breakthrough in our ability to perform translational research and deliver personalized and precision medicine. A multidisciplinary approach in combination with use of novel techniques allows for the extraction and analysis of vast quantities of data even from the volume and source limited samples that can be obtained from human subjects. Continued advances in microfluidics, scalability and affordability of sequencing technologies, and development of data analysis tools have made the application of a multi-omics, or systems, approach more accessible for use outside of specialized centers. The study of alloimmune and protective immune responses after solid organ transplant offers innumerable opportunities for a multi-omics approach, however, transplant immunology labs are only just beginning to adopt the systems methodology. In this review, we focus on advances in biological techniques and how they are improving our understanding of the immune system and its interactions, highlighting potential applications in transplant immunology. First, we describe the techniques that are available, with emphasis on major advances that allow for increased scalability. Then, we review initial applications in the field of transplantation with a focus on topics that are nearing clinical integration. Finally, we examine major barriers to adapting these methods and discuss potential future developments.

## Introduction

**FIGURE 1:**
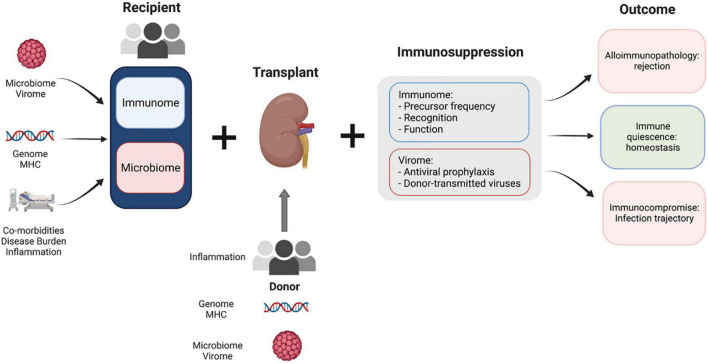
Complex factors play a role in kidney transplant recipient outcomes. Baseline factors at the time of transplantation for both donor and recipient determine the immune micro-environment in the transplant recipient. Modulating factors such as immunosuppression and antiviral prophylaxis moderate the recipient response to this disturbance. The interplay between the fixed baseline characteristics of donor and recipient and modifiable features *(immunosuppression)* determines the outcome for each transplant recipient.

While organ transplantation can provide life-saving therapy, our understanding of the immunology that determines each patient’s trajectory remains immature. The immune response to transplantation is complex, requiring us to consider an incredibly heterogeneous set of characteristics that differ in each recipient-donor pair, including human leukocyte antigens (HLA), comorbidities, prior exposures, and immunosuppressive medications (Figure 1). These baseline characteristics are then modulated by the various medications, type and timing of therapies, and new immune exposures that recipients encounter after transplant. The interplay of these factors will ultimately lead to a trajectory characterized by alloimmune pathology, immunocompromised, or, hopefully, a state of immune quiescence with respect to the allograft and immune readiness for encounter with pathogens.

Although short-term outcomes in renal transplantation have improved dramatically over the past decades, gains in long-term patient and graft survival rates have lagged behind. Late graft failure or premature death from infection or cancer remain major clinical problems, with 10 year graft survival rates hovering around 50% (1). The underlying causes of late graft failure are often complex, sometimes occurring without obvious evidence of contributing immune injury (2–4). Follow-up is less frequent in the long-term, and progressive loss of function is often attributed to scarring, though it can be difficult to clearly discern the cause. Alloimmune injury, including T-cell mediated acute or chronic active rejection and T-cell dependent antibody mediated acute or chronic injury, may be a contributing factor. However, in late graft loss, this is often a mixed picture. Other factors contributing to chronic allograft nephropathy, for example calcineurin inhibitor (CNI) toxicity and BK virus nephropathy, can be amplified by increasing immunosuppressive burden. Non-immune injury from medication toxicity, including CNI, can lead to interstitial, tubular, and vascular injury, and infection, such as BK, can both cause direct injury and lead to subsequent immunopathology. The emphasis on decreasing early acute rejection rates with intense immunotherapy, including the use of T-cell depletion as induction therapy, may contribute to long-term graft failure and/or patient death rates by leaving transplant recipients in an immunocompromised state (5, 6).

Even with relatively moderate immunosuppressive strategies, transplant recipients are particularly susceptible to viral infections, secondary to the specificity of immunosuppressive medications in compromising T-cell mediated immunity (7). This risk has been magnified by the global SARS-CoV-2 pandemic, as transplant recipients have been found to mount suboptimal responses to vaccination (8) and have significantly higher rates of severe infections and hospitalizations (9, 10). While this risk and the risk of other viral infections can be mediated by vaccination prior to the initiation of immunosuppression (11), the landscape of prior immune exposures affects the profile of side effects accompanying immunosuppressive medications and provides an opportunity to further personalize medical therapy (12).

To continue making improvements in long-term outcomes requires us to seek new approaches. From the first era of technical developments in kidney transplantation, we have moved through a second era, highlighted by the introduction of more effective immunosuppression. However, treatment remains largely similar for all recipients, using a protocol with limited variation [cytomegalovirus (CMV) status, panel-reactive antibody (PRA)] and a reactive approach to monitoring drug levels, renal function, viral loads, and DSA. Rapid advances in immune profiling and multi-omics promise a new era of personalized immunotherapy. Personalizing medicine based on patient baseline and response to therapy offers an opportunity to improve long-term outcomes. Baseline and longitudinal biomarkers have the ability to predict downstream events, guiding ongoing risk stratification and personalized management. In addition, biomarkers also yield mechanistic insights that may identify new therapeutic targets for specific patient subsets. While small steps have been made looking at individual markers, the integration of multiple measurements and the use of higher resolution techniques that reduce background signal, by a systems approach, will be necessary to significantly prognosticate and build intuition on the immune system dynamics surrounding transplantation.

In this review we focus on major advances in biological techniques, discuss how these tools are improving our understanding of the immune system and its interactions, and review current applications in the study of transplantation. We aim to break down the alloimmune response to solid organ transplant, with a particular focus on kidney, describe the omics approaches best suited to characterize these components, and introduce key concepts and methods used in the analysis of high-dimensional datasets. We discuss how the application of omics approaches is improving our understanding of the immune system and its role in kidney transplantation. Finally, we describe the main obstacles to adopting systems immunology and discuss potential future developments.

## Systems biology

Systems biology is an area of research that aims to understand the mechanisms of complex biological systems and predict their behavior across scales: molecular-to-organismal (13). This approach allows us to embrace the complexity of a system and the, often degenerate, interactions between its components as a fundamental feature of the network. The immune system is a natural application of the systems approach, with a multitude of components interacting at various levels. Our current understanding of the immune system has been developed through painstaking investigation of individual genes, proteins, and cell types, most commonly in model organisms. Though we owe our fundamental understanding of the immune system to research performed using these traditional methods, further advance requires a shift in perspective.

The global measurement of component features in systems biology allows us to study perturbations in a system without relying on a model organism, avoiding the barrier of translating findings outside of that model. Additionally, considering all components of a system reduces the bias of investigators to focus on specific genes or cell types with prior associations. Finally, incorporating measurements of multiple features for each individual cell brings a deeper level of biological meaning to the function of the network. Considering the enormous complexity of the immune system, with approximately 350 cluster of differentiation (CD) receptors, over 100 cytokines and chemokines, thousands of genes and as many cell subsets (14), the systems approach is a critical strategy for understanding human immunology (15). This approach allows us to use knowledge about the interaction and dynamics of the most varied types of networks (proteomics, genomics, metabolomics, transcriptomics, epigenetics, etc.) to understand a highly complex organism, such as the human body. By considering multiple aspects of disease pathophysiology, the systems approach provides the potential for fundamental new insights into our therapeutic and diagnostic approach to disease (16).

Continued advances in microfluidics, affordability of sequencing technologies, accessibility of high-performance computing and development of data analysis tools have made the application of a multi-omics, or systems, approach more accessible for use outside of specialized centers. In addition, developments in bioinformatics have made it possible to generate integrative models of the immune response, allowing us to measure a cell’s activation state, intracellular signaling pathways, cellular products (cytokines, chemokines, and metabolites), and the genes that encode all of these molecules (15). The implementation of the systems approach has been incredibly successful in the fields of vaccinology, oncology, and infectious disease (17–19). However, perhaps owing to the complexity of data management, modeling, and statistics necessary to wrangle the sheer bulk of data produced in these novel methods, systems methods are just beginning to see applications in many subfields of immunology, transplantation included. Directing focus toward a systems approach in the study of transplant immunology has the potential to advance portions of the field that have been stagnant by traditional methods.

### Systems immunology techniques for characterization of the alloimmune response

In the study of transplant immunology, the key measurable components of the immune system are cells, cytokines and other molecular biomarkers, and cell-free nucleic acids (cfNA) relating to the microbiome, microvirome, or organ donor. Cells can be phenotypically and functionally characterized at the level of surface markers, genome, transcriptome, receptor specificity (for B- and T-cells), or the proteome with the aid of functional assays.

#### Cell surface proteome characterization

The surface proteome has been frequently evaluated by transplant immunologists as an easily accessible external signal of the inner workings of the cell. Flow cytometry, the original single cell method, is capable of measuring multiple parameters, however, the number of features measured is limited by the need for spectral deconvolution. A recently developed technique, cytometry by time of flight (CyTOF), expands the number of parameters measurable by conjugating antibodies to heavy metals rather than fluorescent probes (20). Because CyTOF relies on mass differences detected by time-of-flight measurements, it does not encounter the parameter limitations imposed by spectral overlap as occurs in flow cytometry, rather parameters are limited only by the number of heavy metal isotopes (21). As single cell sequencing methods continue to develop, the surface proteome can now be characterized at the single cell level with more than 100 parameters by using antibodies conjugated to oligonucleotide tags (22). While the cost of single cell sequencing remains prohibitive, throughput is rapidly increasing.

Traditionally immune subsets have been assessed by supervised methods using expert driven manual gating to evaluate T-cells and characterize into subsets by features such as memory or regulatory profiles. However, as the number of measurable markers expand, the combinatorial possibilities increase exponentially. In this situation, manual gating leaves most data unanalyzed. Unsupervised approaches using dimensionality reduction and clustering create new opportunities for insights into these high-dimensionality datasets, the application of which is discussed later.

#### Genomics

From an immunological perspective, the major difference between transplant recipient and donor can be traced to HLA protein polymorphisms. Solid organ transplant recipients and donors most commonly undergo low- or intermediate-resolution human leukocyte antigen (HLA) typing via real time polymerase chain reaction (rtPCR) or antigen specific Luminex-based assays. Alternatively, high-resolution typing with sequencing of the HLA loci can be performed (23). Knowledge of the exact amino acid composition of recipient and donor HLA proteins has led to a variety of approaches toward matching patients at a more precise level, including eplet (24, 25), electrostatic (26), hydrophobic (27), and amino acid matching (28). Perhaps the most widely applied in the United States has been calculation of eplet mismatch burden with use of HLAMatchMaker (24). Calculation of mismatch burden in general, whether via eplets, triplets, PIRCHE score, or an alternative method, have demonstrated association with graft outcomes (29, 30).

Differentiating between recipient and donor genomes is also relevant in the use of circulating cell-free (cf) nucleic acids assays to track complications after transplantation (31). The isolation of nucleic acid from circulating plasma has generated progress in several ways. Most familiar to practicing clinicians is the use of circulating donor-derived cell-free DNA (dd-cfDNA) to monitor for risk of rejection (32). Cell-free nucleic acids also offer opportunity to better understand the pathogenicity of opportunistic infections. Shotgun sequencing of cfNA can allow us to characterize the microvirome and microbiome of transplant recipients throughout the post-transplant timeline, offering the potential to discover novel pathogens (33). Additionally, using epigenomics, the methylation patterns of circulating cfNA can be used to identify the source tissue, thereby isolating the site of damage, whether alloimmune or infectious in etiology (34). Study of cfNA has the potential to create insights into diseases ranging from liver and kidney disease to Alzheimer’s disease and bone marrow transplantation complications (31, 35, 36).

#### Immune cell specificity

With an estimated 10^11^ different T-cell receptors (TCRs) in each individual’s repertoire (37, 38), and few “public” TCRs shared between individuals, the analysis of receptor specificity and similarity is non-trivial. TCR specificity to date has most effectively been determined using HLA tetramers presenting a peptide of interest (39). However, the combination of TCR-HLA restriction and the numerous HLA alleles have restricted progress in this arena to only the most frequently encountered HLA alleles and few peptides (40). Resources such as VDJdb have begun to compile TCR sequences in combination with their defined antigen specificity (41).

Currently, TCR sequencing is dominated by commercial ventures who have optimized methods for capturing and amplifying the repertoire of a sample. Scoring TCRs by gene usage and amino acid similarity has been successful in predicting TCR specificity after training on datasets with known specificity or pathologic relationships (42–44). As with surface proteomics, TCR sequencing by single cell sequencing methods is becoming more affordable and higher throughput. Single cell sequencing captures both alpha and beta chain of the TCR, rather than solely the beta chain as in most bulk TCR sequencing, not only providing more information on cell specificity, but also creating the potential to artificially express the TCR in a model system (45).

#### Single cell sequencing

Perhaps the most revolutionary technology to drive discovery in systems immunology is single cell sequencing. Cells can be captured by multiple methods, however, the most broadly implemented method, due to its scalability, is droplet-based cell isolation. Each cell is captured in a droplet emulsion along with a gel bead containing a unique molecular identifier (UMI) (46–48). Reverse transcription is performed inside of the droplet so that all complimentary DNA is labeled with the UMI. The droplets can then safely be disrupted, and cDNA recovered for amplification by polymerase chain reaction (PCR), library preparation, and sequencing. In addition to the capture of paired chain TCR, another added benefit is the ability to analyze surface protein expression, with cellular indexing of transcriptomes and epitopes by sequencing (CITEseq). To do this, before single cell isolation, cells are stained with antibodies conjugated to oligonucleotide tags. When cells are subject to reverse transcription, the antibody tags are captured and linked via UMI as well, allowing surface markers to be retained to the individual cell (49).

### Comprehensive overview of state of the art

**FIGURE 2:**
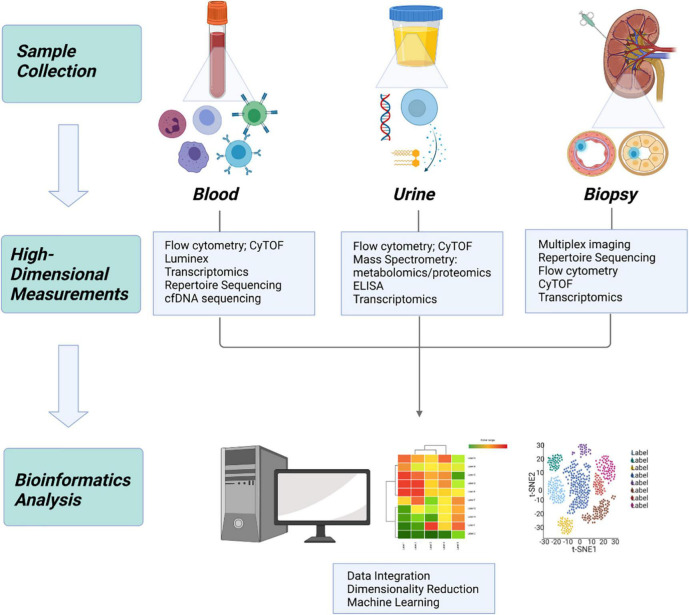
Application of systems immunology to transplantation. In kidney transplantation, samples can be obtained from 3 primary sources: whole blood, urine, and allograft tissue. High dimensional measurement of each sample type provides a unique source of information, which can be integrated using bioinformatics to build a more complete understanding of the biologic system.

Though applications in kidney transplantation have been limited, authors contributing to this literature have overcome significant hurdles and these results have laid the groundwork for further additions to the field (Figure 2).

#### Biopsy omics

Recent study of kidney transplant biopsies have been heavily weighted toward identifying high-throughput transcriptomic signatures associated with specific disease pathology and using those signatures to improve early discrimination in diagnosis. Using microarray analysis, Halloran found that the antibody mediated rejection (AMR) rejectome is characterized by endothelial transcripts, reflecting angiogenesis secondary to endothelial injury. In contrast, many IFNy-induced genes are seen in both acute T cell mediated rejection (TCMR) and AMR, and acute kidney injury (AKI) transcripts are found to indicate disease progression across different forms of injury (50–53). This work notably led to the discovery of the C4d-negative AMR phenotype (54, 55). Halloran’s work led to appreciation of increasing rates of AMR over time, along with the recognition that AMR is strongly associated with graft loss, in contrast to TCMR (56, 57). Biopsy transcriptomics has also been applied to critique pathologic diagnoses of rejection (50, 58). However, unsupervised clustering (see Section “Data science capabilities”) with large gene panels has been unable to discriminate pathologies well due to the similar gene expression profiles in many forms of graft injury, requiring a supervised approach to achieve higher resolution of groups (57). While possible applications are numerous, microarray transcriptomics has been modified for an early incorporation into clinical care as a predictor of graft stability and future course, notably by the “molecular microscope diagnostic system (MMDx)” (59, 60).

#### Peripheral blood omics

While the early diagnostic discrimination provided by biopsy transcriptomics is promising, it still requires an invasive procedure for tissue sampling. The transcriptomics field has inspired much optimism toward developing a non-invasive bioassay capable of detecting rejection. At the beginning of the last decade, the kSORT assay was developed, using quantitative polymerase chain reaction (qPCR) to measure expression of a set of 17 genes identified (out of a panel of 43 examined) as discriminatory of rejection. While the multicenter AART study (61) initially validated the test as predictive, a recent application in a large retrospective cohort found no diagnostic value (AUC 0.51) (62).

Proteomics of peripheral blood has also been appealing because of the relatively straightforward transition to clinically relevant assays with Luminex platforms (63) or flow cytometry. Importantly, sequencing of cfDNA isolated from patient plasma offers the opportunity to characterize the microvirome of this immunosuppressed patient population. While commensal organisms have been tied to host health in the context of many disease states, the relatively targeted suppression of T-cells by transplant immunosuppressants makes these patients particularly susceptible to viral infection. Furthermore, an analysis of heart and lung transplant patient microviromes demonstrated the correlation between microvirome state and risk of rejection (33). Another assay of cfDNA that has reached a more widespread level of application is detection of donor-derived cfDNA (64, 65). While dd-cfDNA has the ability to detect graft injury, it has not yet achieved great success in differentiating pathologies. One of the biggest challenges in the field has been the design of studies to determine the most suitable role for these techniques in clinical practice.

#### Urine omics

In kidney transplantation, the ability to obtain a “liquid biopsy” is enhanced by the proximity of not only blood, but an additional biofluid, urine. The study of urine for possible biomarkers of rejection has received a lot of attention and spans transcriptomics, proteomics, metabolomics, and genomics approaches. Early work in analysis of mRNA from cellular debris in transplant patient urine samples demonstrated association between transcripts for mediators of cytotoxicity and acute rejection (66–71). Over the past decade, the work has shifted in the direction of a systems approach. Clinical trials in organ transplantation (CTOT-4) investigators identified a panel of 3 transcripts associated with rejection, with a signature detectable up to 20 days prior to biopsy-proven diagnosis (72). A later analysis used a panel of 26 genes to identify a linear combination of 5 transcripts capable of discriminating TCMR from AMR (73).

Urine metabolomics and proteomics have followed a similar trajectory. Initial studies using enzyme-linked immunosorbent assay (ELISA) or similar solid-phase bead-array assays identified associations with cytokines of interest (74–76), though applying individual urine cytokine detection as a threshold for biopsy was generally found to be insufficiently sensitive (77). A more conservative approach proposed by the CTOT-1 consortium is to consider urinary protein levels (in this case CXCL9) in adjusting immunosuppression (78).

The more recent escalation to higher dimensionality measurement via large-scale antibody arrays and multiplex beads assays began to allow for an unsupervised approach to urine biomarker discovery (75, 79). The field has since shifted to a true systems approach driven by Minnie Sarwal et al. at UCSF, who have used mass spectrometry to perform shotgun high-throughput proteomics (80–83), which has been successful in creating panels that discriminate between pathologies (84) and have been translated into a spot based diagnostic assay (85). Contributions from other labs are leading toward providing resolution between AMR and TCMR (86, 87).

A subfield that has largely been neglected in application to urine biomarkers is measurement of the surface proteome. Several early papers presented immune cells in the urine as a marker of rejection (68, 88–90), though this was not pursued in follow-up until very recently (91). This is a promising approach that may benefit from increased dimensionality, using an unsupervised approach, and incorporation into a multi-omic characterization along with urine transcriptome and metabolomics. Both surface proteomics and free fluid omics share a common limitation due to the dilute concentrations encountered in urine (92). However, surface proteomics also relies on live cells in order to avoid non-specific binding, a significant additional limitation.

### Ready for prime time

#### Cell-free DNA

While a number of insights can be drawn from circulating cfDNA, the application that has more recently gained traction in the clinical practice realm is monitoring dd-cfDNA. To our knowledge, three companies have commercially available tests monitoring dd-cfDNA (93). These tests use next-generation sequencing of cfDNA to detect single nucleotide polymorphisms (SNPs) that can distinguish between donor and recipient cfDNA (94), allowing for readouts of the absolute quantity and relative proportion of dd-cfDNA. Early interest in dd-cfDNA gained momentum after the DART study (Circulating Donor-Derived Cell-Free DNA in Blood for Diagnosing Active Rejection in Kidney Transplant Recipients) demonstrated that a threshold of only 1% dd-cfDNA was useful in discriminating active rejection (95). While the limitations of the test were clear, as observed in the notably lower levels of dd-cfDNA detected in TCMR when compared to ABMR, this still represented a sensitive, non-invasive test with the potential to provide more specific guidance on the need for invasive biopsy than that obtained from serum creatinine.

Further study with serial observation of dd-cfDNA in a large cohort of kidney transplant recipients in the ADMIRAL study (Assessing Donor-derived cell-free DNA Monitoring Insights of kidney Allografts with Longitudinal surveillance) identified a cutoff of 0.5% as a surrogate marker for immune quiescence and preservation of eGFR (32). This study importantly demonstrated the utility of dd-cfDNA not only in discriminating active rejection but also in predicting future allograft function (96). However, as dd-cfDNA does not provide the diagnostic granularity to replace the need for biopsy, its most promising application is in early detection of allograft risk allowing for more frequent monitoring, or in evaluating patients’ appropriateness for reduction of immunosuppression.

Studies of dd-cfDNA outside of these initial trials have led to variable outcomes and an overall lack of consensus regarding the most appropriate application of the test. While studies have generalized that patients with higher dd-cfDNA experience higher rates of rejection, the ability to accurately predict adverse events among these patients is still lacking (97). This is because dd-cfDNA is elevated in a multitude of pathologies and lacks the ability to distinguish among infectious or immunologic etiologies of graft injury (98). In fact, some studies suggest that among low-risk kidney transplant recipients, the benefit provided from early dd-cfDNA monitoring may only be marginally better than current clinical management (99). For now, dd-cfDNA has found a niche in the surveillance of high-immunologic risk patients for early detection of a threatened allograft and an opportunity to alter immunosuppression regimens. However, the cost-effectiveness of these strategies must be weighed carefully, as the cost of each dd-cfDNA assay is not trivial (93).

#### Eplets

Eplets, a set of polymorphic amino acids within a 3-angstrom radius, represent the smallest functional unit capable of determining antibody specificity. The tabulation of eplet mismatch between HLA molecules can be used to quantify the alloimmune burden between recipient and donor HLA. Though the study of eplet disparities continues to evolve rapidly as methods to handle the varying immunogenicity of particular eplets are developed, the underlying principles of eplet mismatch as a correlate to clinical outcomes has been strongly established (100–104). Eplet mismatch clearly has a role in risk stratification of transplant recipients during the post-operative period. Wiebe et al. (105) demonstrated that patients with a lower eplet mismatch burden are better able to tolerate lower tacrolimus troughs without developing DSA, a finding that has been reproduced in multiple independent cohorts (106, 107). As these studies reinforce, understanding of eplet mismatch burden will enable personalization of immunosuppression reduction after transplantation.

Prospective matching using eplet disparities has also received a considerable amount of attention, primarily in the pediatric population and by the Canadian transplant community. The Genome Canada Transplant Consortium (GCTC) performed a study using simulations of targeted eplet matching and demonstrated that, in the Canadian population, perfect identity at the class II loci could be obtained with a waiting list size of approximately 250 transplant candidates. The GCTC is currently underway in evaluating the feasibility and repercussions of implementing epitope-matched allocation at the national level (108). In pediatrics, the importance of a close match between donor and recipient is magnified, as the likelihood of re-transplantation is significantly higher in this population. In recognition of this, a small prospective study examined the outcomes of transplant recipients who were matched with an additional exclusion criteria for potential donors with a high eplet mismatch burden (109). Patients who were transplanted within the additional donor exclusion criteria experienced a lower rate of DSA at 1 year follow-up. While this is a promising result, the complexity of deceased donor organ allocation means that further examination of the outcomes, equity, and systemic effects of epitope matching are needed before broader application can be considered. In contrast, eplet matching in the context of living donor paired exchange has begun to gain traction at a number of centers (104).

However, viewing eplets as a count metric with a singular weight is an oversimplification of the biology the method aims to represent (110). In particular, DQ and DR mismatches appear more likely to lead to DSA and kidney graft failure (111). To compensate for this difference in biological importance, Wiebe et al. (105) developed single molecule eplet mismatch scoring, which considers only the mismatch of DR and DQ loci, and uses molecule-specific counts. The field continues to seek further refinement, by considering the varying immunogenicity of specific eplets in terms of association with antibody formation (110, 112, 113). However, early studies suggest that immunogenicity of eplets may vary depending on the transplant recipient population demographics (114–116). This may reflect the importance of T-cell biology to the outcome of AMR. One tool, PIRCHE, outputs a score reflective of the ability of recipient HLA to present peptides from donor HLA (117). This weighting of the role of the indirect pathway has been associated with transplant recipient outcomes (118). While this has not yet been evaluated, it could be hypothesized that the varying immunogenicity of eplets across demographic subsets may be related to the ability of recipient HLA to present relevant peptides and recruit T-cell help to the antibody response.

Although the original eplet matching software (HLAMatchMaker) represented a major breakthrough in this field, it suffered from lack of reproducibility and a high barrier to use. Recently, our group published a high-throughput tool, hlaR, available as a web application (119) and on CRAN (120), that applies HLAMatchMaker reference tables and logic in a format that is accessible to a broader user base (121). However, without high-resolution HLA typing, the accuracy of these tools are constrained by the imperfections of the method of imputation used to convert HLA typing from low to high-resolution (122, 123).

#### Digital pathology

The term digital pathology was created after the introduction of digital images generated by microscopes and represents the use of technology to assist in the creation, sharing, exchange of information and analysis of tissues and cells performed by the pathologist (124). The latest technological advances including super-resolution and single-molecule imaging associated with sequencing approaches have transformed pathology, allowing us to better understand cell types, interactions, and heterogeneity in complex tissues. Most importantly, this has enabled spatial transcriptomics, the ability to extract spatially resolved molecular information from tissue biopsies, which was named Nature’s 2020 Method of the Year (125, 126). Spatially resolved omics can be divided into two main categories: one using multi-omic analysis of microdissected tissues followed by computational reconstruction of spatial data and another using *in situ* hybridization or single cell barcoding before sequencing and analysis.

In transplant biology, graft rejection diagnostics have essentially been based on clinical and histological criteria. While histologic diagnosis remains the gold standard, it suffers inconsistencies due to the variations in histologic grading and considerable interobserver disagreement. In an attempt to align and standardize histological diagnostics for transplanted organs, the MMDx was developed (59, 127, 128). MMDx is a microarray-based gene signature platform that evaluates kidney allograft biopsies to predict graft injury and rejection. MMDx evaluates the gene signature assigned to each particular biopsy region by combining molecular measurements with machine learning classifiers.

At the last Banff meeting in 2019, the Banff Molecular Diagnostics Working Group suggested the use of transcriptomics in combination with immunohistochemical assays and conventional pathological evaluations for the diagnosis of transplant rejection. The recommended Banff Human Organ Transplant (B-HOT) NanoString panel includes 770 genes, covering the most pertinent genes related to rejection, tolerance, toxicity, viral infections, innate and adaptative immune responses (129). However, bulk ribonucleic acid (RNA) sequencing of graft biopsies may not capture the focal nature of acute rejection. Multiplex spatial biology techniques with molecular resolution up to single cell level may be the future of rejection analysis in transplant biopsies (130–135). Recently Salem et al. (136) showed that the NanoString whole exome GeoMX Digital Space Profiling platform can be used to study the transcriptional profile in different regions of acute rejection biopsies. Another use of digital pathology in transplantation has been the use of artificial intelligence for automated image analysis of immunological synapses and cell activation in biopsies stained by multiplex immunohistochemistry (137). However, the high cost and the need for a fully integrated multidisciplinary group to develop these spatially resolved omics technologies are today the biggest challenges prohibiting the routine employment of these technologies, especially for clinical diagnostics.

### Emerging technologies

#### T-cell receptor sequencing

Most extensive study of the human T-cell repertoire has focused on response to infectious pathogens, notably CMV, Lyme disease, and COVID-19. In a landmark study, Emerson et al. (138) performed immunosequencing of 666 healthy humans with known CMV status and HLA class I type. These 90 million TCR beta chains opened the door for the application of technology and bioinformatic pipelines to guide TCR analysis and demonstrated that TCR repertoire could be used to distinguish individuals by characteristics of their immune system such as viral exposure or HLA type. Public CMV-associated TCRs, or those found in multiple individuals correlated with CMV serostatus, were identified. Examples of these public TCRs were experimentally confirmed to indeed be specific for CMV epitopes in HLA molecules expressed by the blood donor.

Public TCRs, sequences that are statistically more likely to appear across multiple individuals’ repertoires, may occur because of the biologic advantage incurred, because of the redundancy of the genetic code, or because of the entropic favorability of specific rearrangements (139, 140). The presence of these public sequences allows for the use of TCRs as biomarkers of prior exposures. Though the presence of even public TCRs can be subject to the stochastic nature of recombination, generalization of the specific TCR into a meta-clonotype of biosimilar, quasi-public TCRs likely to recognize the same epitope can provide a more rigorous search metric to fully characterize the T-cell response (141). Quantifying the presence of these antigen-specific TCRs by frequency in the overall repertoire, which has been termed breadth for unique clones and depth for total templates, may inform the quantity and quality of the anti-pathogen T-cell response. Following CMV, this approach was later applied successfully in both Lyme disease and Epstein-Barr Virus (EBV) (142, 143).

These expanding databases of TCRs and cognate antigen have allowed for additional insights into the TCR repertoire and implications for protective immunity, most recently with SARS-CoV-2 (41). Just as CMV naïve transplant recipients have markedly distinct patterns in TCR sequences when compared to CMV seropositive transplant recipients (144), this concept has been extrapolated to the application of SARS-CoV-2 exposure analysis. Prior exposure and T-cell mediated immunity can be inferred by comparing an individual’s TCR repertoire against a classifier developed from a database of known public SARS-CoV-2 associated TCRs (145, 146). This methodology has recently been expanded to diagnosis of other vector-borne disease and has the potential to be applied to innumerable viral pathogens (143). TCR repertoire has proved to be useful in evaluating response to vaccination, and may be a useful adjunct in screening patients for baseline protective immunity prior to transplantation to guide prophylaxis or duration of therapy (147, 148).

An exciting potential application in transplantation led by M Sykes has coupled TCR sequencing with alloimmune mixed lymphocyte reaction (MLR) to identify the donor reactive TCR repertoire (149). The donor reactive TCR repertoire can be used to assess the specificity of graft infiltrating lymphocytes during rejection, as well as to monitor for the development of tolerance and deletion of alloreactive clones after liver transplantation or chimerism induction (150, 151). While this work is in early stages, the alloreactive TCR repertoire may be useful as a diagnostic tool, predictive of rejection with expansion or induction of tolerance with contraction. However, the sensitivity and required sampling depth for these assays need to be explored and characterized. Cell based assays are cumbersome and may have limitations in reproducibility, but nonetheless offer exciting potential applications.

There are numerous, complex forces involved in shaping the alloimmune T-cell response. Alloreactive TCR repertoires vary based on widely variable recipient HLA alleles driving T-cell selection during development, the ability to indirectly present donor non-self peptides, the variety of self-peptides that may be presented in the direct pathway on donor HLA, and even random chance (152). Though this requires significant work for each candidate transplant donor-recipient pair, it does provide a useful diagnostic, though not therapeutic, tool that can track alloreactive TCRs post-transplant to determine the risk of rejection (153). Ideally, understanding each component involved in shaping the alloreactive TCR repertoire could allow for computational development of an alloreactive-specific TCR depletion method. Significant work has been done in determining non-self peptide ability to bind recipient HLA (117), but our knowledge of the immunopeptidome, and our ability to predict the cognate TCRs to specific epitopes is still nascent (154–157).

#### Immunopeptidome

Major advances in our understanding of the immunopeptidome have been made in recent years due to improvements in wet lab and computational methods. T-cells are known to recognize peptides when presented on HLA proteins; the HLA-peptide complexes that are recognized by allospecific T-cells make up the immunopeptidome. CD8 T-cells recognize HLA-I presenting peptides that are 8–10 amino acids long. In contrast, for CD4 T-cells, peptides are presented on HLA-II and are longer, most often 15mers, due to the open binding pocket of HLA-II. Peptides eluted from HLA have been identified using mass spectrometry to define both the self-peptidome and pathogen-related peptidomes. The large datasets of these peptides and their presenting HLA proteins led to the identification of allele-specific binding motifs. Machine learning prediction algorithms, such as netMHCpan, have been trained using these elution datasets as well as other large datasets containing peptide binding affinity (158). These algorithms, which were recently used to rapidly predict SARS-CoV-2 epitopes, compress the time required to develop tetramers and characterize the T-cell response from years to months.

While application in transplant is in the early stages, the two components of response to understand are the donor HLA-I presented peptides on the allograft and the allogeneic peptides presented on recipient HLA-II. Elegant work by Son et al. (154) used a mouse model to identify immunodominant peptides contributing a large portion of the direct alloresponse, suggesting the importance of the peptides presented by donor HLA-I. Additionally, the viral peptidome can lead to an alloimmune response through heterologous immunity. For example, a T-cell that recognizes an EBV peptide in the context of HLA B8 can be cross-reactive against B44 with a self-peptide, leading to an alloimmune response (159–163). However, the self HLA-I immunopeptidome is vast, with the ligandome for each allele measured in the thousands or tens of thousands. The component of the alloimmune response due to indirect allorecognition, while still complex, is more readily trackable. The indirect immunopeptidome is presented by recipient HLA-II and is limited to polymorphisms between donor and recipient (most often HLA), the basis for PIRCHE II.

In studying the indirect immunopeptidome, we aim to understand the contribution of HLA class II peptide presentation in recruiting T-cell help for the alloimmune response. PIRCHE II is a sophisticated implementation of this approach that generates a score for each donor antigen in the context of each recipient HLA class II allele, representing the number of unique, non-self, core sequences from the donor antigen that are expected to bind to and be presented by the recipient HLA (117). In just the past few years, PIRCHE II scores have been associated in studies of kidney, lung, cardiac, and liver transplant recipients with increased risk of DSA and TCMR (164–167).

While there is likely utility in using PIRCHE II for post-transplantation risk stratification, an exciting possibility for clinical use is in evaluating potential donors for transplant candidates with a prior sensitizing event. Evaluation of shared T-cell epitopes in conjunction with cPRA can help to predict the risk of early DSA formation in sensitized candidates (168). Population level studies have been used to construct a tool that allows clinicians to evaluate the distribution of PIRCHE II scores for a given recipient across their center’s donor population, providing context for the level of T-cell epitope match seen with a given donor offer (118).

Though PIRCHE II is rapidly progressing toward the clinical realm, the immunopeptidome as it relates to HLA class I peptide presentation is equally exciting, albeit less developed. The CD8 alloimmune response is most likely responding to commonly occurring peptides in the context of non-self HLA class I. In this case, the overall peptidome is essentially constant between individuals, with the non-self class I HLA driving the recognition of peptide-HLA as foreign. Recent large scale projects are undertaking the mapping of the human ligandome across organs and HLA types (155, 169). As this data evolves, we are optimistic that immunodominant peptides in the alloimmune response will arise. The identification of immunodominant peptides, both for class I and class II HLA, opens possibilities for tolerogenic therapy, with oral tolerance of class II peptides (170) or tetramer mediated depletion of cognate T-cells (171). Likewise, more comprehensive mapping of the viral peptides involved in the immune responses to BK, CMV, and EBV may facilitate new assays to better understand transplant recipient protective immunity.

### Data science capabilities

Our entrance into the era of “big data,” with increases in the volume, variety, and velocity of data generation, requires new approaches to harness the power of all the available data. With data coming from multi-omics, electronic health records, patient recorded devices, and blood tests, we have only begun to skim the surface of the exciting opportunities beginning to arise. Moving forward, studies will require the integration of multimodal data sources, which requires more complex analysis methods (Table 1). Bioinformatics offers multiple approaches including network analysis, integration of longitudinal data, and machine learning that is supervised, unsupervised, or semi-supervised. Depending on the methods employed, analysis of these high-dimensional datasets can lead to hypothesis generation, or prediction models which range from explanatory to “black box” depending on the level of supervision used. To ensure reproducibility of results, these analysis methods need to be developed in the context of data processing pipelines so that local and personal data can be analyzed and compared to public datasets. Ideally, these methods should also be built into graphical user interfaces to allow interface between clinicians, immunologists, and computational biologists. These new opportunities and their accompanying challenges highlight the need for new methods and new collaborators in the transplant community.

**TABLE 1:** Bioinformatics tools and review articles for some of the key aspects of systems immunology that are most relevant to transplantation.

Analysis	Key bioinformatics tools	Review articles
TCR/BCR repertoire	TCRdist3 (141), CDRdist (44), GLIPH2 (42), Immunarch (183), VDJPuzzle (184)	Rosati et al. (185), Teraguchi et al. (186)
Flow/mass cytometry	flowCore (187), cytoExploreR (188), FlowSOM (189)	Saeys et al. (190)
Single cell RNA seq	Seurat (191), Scater (192)	Andrews et al. (193), Luecken (194)
Metagenomics	Metaspades (195), kraken2 (196), viral_ngs (197)	Quince et al. (198)
High-resolution HLA	HLAMatchMaker (24), HLA-Emma (28), hlaR (121)	Sahin et al. (199)
Trajectory analysis	Monocle (181), slingshot (200), GPfates (201)	Saelens et al. (202)
Network analysis	GeneMANIA (203), SNF (204), iGraph (205)	Jiang et al. (175)
Multi-omic integration	iClusterPlus (206), PARADIGM (207), MOFA (208), iOmicsPASS (209), STATegra (210), xMWAS (211)	Subramanian et al. (212)
Differential expression	MAST (213), limma (214), DESeq2 (215)	Costa-Silva et al. (216)

#### Dimensionality reduction

Data generated from sequencing requires significant processing before analysis (Table 1). The processing required varies based on the type of data generated, but the overall goal is to only carry forward data that significantly differs between samples. Specificity data results in TCR or B-cell receptor (BCR) sequences, which may be broken done by V, D, and J regions. Often, this data is simplified to a V gene, a J gene, and the complementarity determining region 3 (CDR3) sequence, which is the most variable portion of the sequence. While unique TCRs are still distinguished by their CDR3 region, this simplification greatly reduces the computational effort required to compare TCRs to one another.

With transcriptomic data, sequencing results are used to determine gene expression levels by cell (for single cell methods) or by sample (for bulk transcriptomics). Although users can manually determine genes of interest to predetermine the focus of the experiment, a less biased approach is variance selection. Rather than considering the expression of every single gene in comparing cells or samples, first variance analysis is performed. If gene expression has minimum variability across the samples, that feature can be dropped with minimal loss of discrimination between samples. This has the effect of reducing variables which enables projection of the data for visualization, as in primary component analysis (PCA), t-distributed stochastic neighbor embedding (tSNE), and uniform manifold approximation and projection (UMAP).

#### Machine learning

**FIGURE 3:**
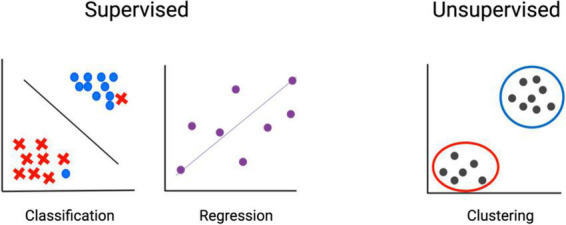
Machine learning–fundamental concepts. In supervised methods, input data is already classified, and machine learning is used to determine associations. In unsupervised methods, input features are used to determine classification.

While experimental design results in some unavoidable level of confirmation bias, machine learning aids in removing preconceptions about the importance of specific cells or genes and considers all features regardless of pre-ascribed ideals. There are two basic branches of machine learning: supervised and unsupervised. Supervised machine learning is applied to data that is already categorized. Applying machine learning to this data is essentially using it to train a model, similar to performing linear or logistic regression. The model built reflects the importance of input features in determining the categorization of each object. The trained model can then be applied to uncategorized testing data and used to assign a classification based on input features. In contrast, unsupervised machine learning is used to sort data that has no pre-assigned classification. Data is input, and the similarity among features is used to categorize objects (Figure 3). This is most frequently implemented by clustering, which is useful both for identifying the presence of distinct populations and for assessing the presence of populations with a high level of similarity. We won’t address the intricacies of clustering approaches here but refer the reader to this recent review (172).

**FIGURE 4:**
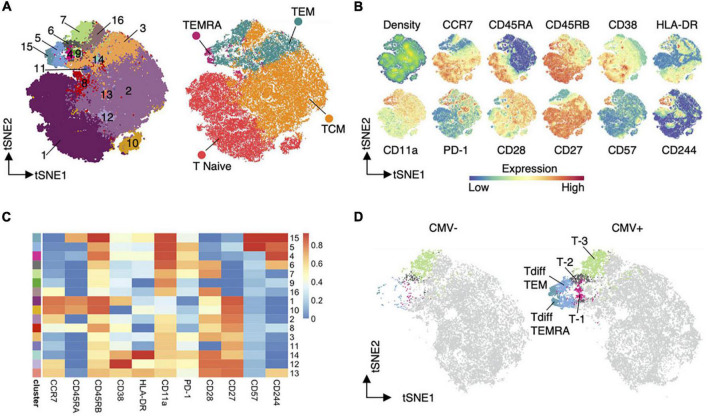
Dimensionality reduction (144). An example of dimensionality reduction use to display the results of unsupervised clustering. Heatmaps **(C)** display the representation of input features in each cluster. While TSNE graphs allow visualization of the distribution of clusters or cell types **(A)**, input feature **(B)**, and detection of differences in cluster frequency between sample types **(D)**.

The most suitable method for analysis varies based on the features of the data. Single cell methods create datasets where the number of features far outnumber the samples. On the contrary, analysis of cell surface markers from flow cytometry or CyTOF, despite increasing discrimination with improved technology, generates datasets with numbers of samples (cells) that are orders of magnitude greater than the number of features measured. Cells are categorized into clusters based on the profile of markers or genes they express. Clusters can then be visualized in several ways. Multiple approaches aim to improve on the visual representation of data including PCA, tSNE, (173) (Figures 4A,B), and UMAP (174). Though difficult to visually interpret, heatmaps simultaneously convey the expression of all markers used in clustering (Figure 4C). All of these are dimensionality reduction methods, which are helpful for data input into models and for understanding model or clustering output by allowing visualization and comparison (Figure 4D).

**FIGURE 5:**
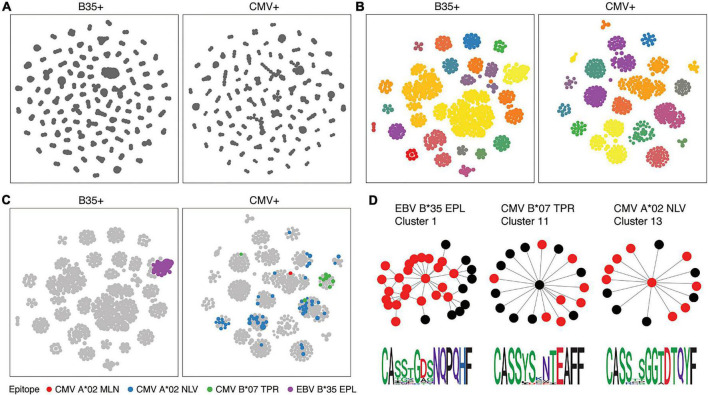
T-cell receptor (TCR) similarity analysis (217). TCRs can be grouped in terms of sequence similarity **(A)**. Connected components can be identified by network analysis **(B)**. Known specificities can be overlaid on similarity networks **(C)**, and motifs inferred **(D)**.

For TCR and BCR sequencing, beyond assessing the presence of clonality, cells can only be grouped after calculating a measure of similarity between the CDR region and V/J gene segments used (Figure 5). There are a number of approaches, ranging from user-friendly GLIPH2 to algorithms that also provide a measure of similarity in the form of a Euclidean distance between each receptor and comparator, such as TCRdist and CDRdist (42–44). This “distance” is measured by sequence alignment, using amino acid similarity scores to optimally align CDR3 sequences. Aligned sequences are used to sum the concordant and discordant positions in each TCR, and distances can then be used to find clusters of similar TCRs.

#### Multi-omic data integration

Multi-omic data can be acquired in two ways. The most approachable method is single-cell sequencing. An alternative method is performing single omic experiments on parallel samples. The integration of single cell data, while still not computationally insignificant, is logically trivial. Each sequencing read has a unique identifier tying the read to the cell it originated from. The unique identifier is the same across the cell surface library, VDJ library, and gene expression library, which allows reads to be integrated across measurements. The integration of single omic data is much more theoretically complicated and generates a less rigorous result. However, the required sample input quality for single cell sequencing is so high that many applications will likely continue to rely on single omic bulk measurements while cost and scalability improve. The current repositories and continued production of this data have encouraged the development of methods to incorporate the results from multiple methods performed on parallel samples from the same subject.

The various tools surrounding multi-omic data integration are targeted toward the user intention. Most frequently, this falls into two categories: disease subtyping or biomarker detection. The ability to perform one implies the capacity to extrapolate the other, but the number of available integration tools allows users to pick one suited to their needs. Methods can simplistically be understood by the timepoint at which data from single-omic datasets are combined. There are two basic options-combine datasets early or late.

Methods that combine datasets early can suffer bias if one omics source has many more features than another. Steps for covariance detection and normalization of datasets to the same scale are required prior to data analysis. Regardless of the method of regularization, early dataset integration is subject to lose the differing weight of importance attributable to each omics source dataset. However, once datasets are satisfactorily combined, clustering and regression on clinical outcomes of interest can be performed in a relatively straightforward fashion, without much difference from a single omic dataset.

A late integration means that each source omic is clustered and analyzed individually before combining datasets. In this approach, each single omic dataset is analyzed to generate its own pattern of associations. The networks generated are subsequently brought together (“fusion analysis”) to determine consensus signatures. The benefit of this approach is the ability to draw conclusions from individual data sources before combining datasets. Building additional perspective on the significance of specific features from the initial datasets can help inform integration steps. Each dataset is ascribed “local” effects, and integration of the datasets allows each omic to be ascribed a weight in determining the “global” effect (pattern fusion analysis).

#### Additional methods

Network Analysis contributes an additional layer to this analysis by incorporating data on the biologic connectivity between components. For example, the known relationships between transcription factors and the proteins they moderate, as well as genes and the proteins to which they are translated, can be used to capture the biological ties between different levels of omics data. In a network, also called a graph, the components being analyzed are visualized as nodes, and the associations between components are edges or vertices (175). Building a network from omic results allows for analysis of data from a topographical approach that captures information in a more visually digestible manner. Nodes can be evaluated for “degree,” a measure of the number of edges extending from the node, or “betweenness,” the frequency at which a node appears in the shortest path between any two given nodes (176, 177). A weighted network can also provide information on the strength of connection between two nodes. There are a number of tools to help researchers use network visualization and analysis to further understand their work, including cytoscape, innateDB, and human disease network (178–180).

In addition to capturing cell states and interactions at a single point in time, we are interested in understanding the trajectory of cell states and the behavior of these networks over time. Collection and analysis of multiple longitudinal samples is a critical component of building this knowledge, but a significant amount of information on cell states and trajectories can be extracted from single cell RNA sequencing (scRNAseq) data by examining cells in pseudotime.

Understanding cell trajectories in scRNAseq data enhances our ability to identify regulatory genes differentially expressed both along and between pathways (181). Pseudotime, or trajectory inference, assigns cells a position along a trajectory from one state to another. The large number of trajectory inference tools can be subdivided by whether the topology is predefined (linear, bifurcating, etc.) or inferred from the data. Inferring topology allows a more complex trajectory to be defined, but as predefined topology can underestimate complexity, inferred trajectory methods can overestimate the complexity of biological networks with simple trajectories. While single cell data provides a particular advantage in the application of pseudotime, for experiments with single omic data, temporal analysis is equally important. The differences in patient immune system composition at baseline can be used to predict responses to perturbations in the environment, such as vaccination (182).

## Discussion

The rapidity of advancement in systems immunology is difficult to overstate. Microfluidics is perhaps the most striking example, where single cell capture has quickly scaled from a cost-prohibitive technique limited to a few hundred cells into a technique that captures tens of thousands, soon millions, of cells and can be performed by individual labs outside of specialized centers with minimal additional training. Many features of single cell technology are evolving–extensions of single cell proteomics, the incorporation of spatial data, ability to formalin fix cells, functional assays, parallel sorting. These will only expand the relevance of a multi-omic approach to our field.

While bioinformatics tools are rapidly advancing in capability and use, the most important advancements are in making such massive scales of data analysis approachable to immunologists not trained in computer science. As the initial generation of samples and ultimate derivation of biological relevance from results falls to immunologists, it is a huge advantage for those researchers to be able to interact with multi-omics data regardless of their familiarity with the command line. In parallel to improved user interfaces, bioinformaticians are developing reproducible pipelines that allow users simplified input and output, reducing decision making and improving standardization. Though too many to list, bioinformatics tools for every facet of the field are improving in terms of computational efficiency and accuracy.

The result of this effort is that several biomarkers are beginning to cross the margin into the clinical realm. While biomarkers are not yet able to replace standard of care procedures, further application will allow us to find an initial role that can be expanded in the future. Urine omics have natural potential in screening patients to determine the need for allograft biopsies. Molecular diagnostics of allograft biopsies may lead to more accurate diagnoses and clarify the interpretation of those with borderline presentation or the appearance of mixed pathologies. Baseline early peripheral blood omics and high-resolution typing can allow us to individualize immunosuppression regimens and prophylaxis more effectively.

Though the benefits of applying systems immunology to transplantation are numerous, there are several limitations to the multi-omics approach in its current form. As the need for data analysis methods is recognized, multiple techniques and tools have been developed in parallel for similar applications, making it difficult to compare results between studies. Standardization of methods and documentation for reproducibility will be critical moving forward. In addition, the systems approach, particularly in application to human subjects, is often applied in an exploratory manner, with hypothesis-generating results. Follow-up study in a targeted manner is frequently required to confirm these initial findings. Additionally, the breadth of knowledge required to integrate findings from multiple techniques can require a high-functioning multidisciplinary team, which can be difficult both to identify and to train.

While the accumulation of knowledge and skill to incorporate these multiple measures into outcome prediction is no small feat, it is the most likely route to success. A single biomarker, despite the utmost care taken in its selection, captures only a single dimension of a patient, who in reality is the most complex network of networks. The future of outcome prediction for transplant recipients lies in our ability to integrate multi-omic data. It is only with more accurate outcome prediction that we can continue to move toward personalized transplant care, using patient baselines and early profiles to tailor immunosuppression, prophylaxis, and follow-up.

## Author contributions

AJ and CL devised the project and conceptual framework. AJ developed the initial draft of the manuscript. JS and SK provided contributions to the manuscript and edited the draft of the manuscript. All authors contributed to the article and approved the submitted version.
